# Effects of Ischemic Post-Conditioning on the Expressions of LC3-II and Beclin-1 in the Hippocampus of Rats After Cerebral Ischemia and Reperfusion

**DOI:** 10.1515/biol-2019-0020

**Published:** 2019-07-10

**Authors:** Liquan Huang, Zizhuo Liu, Lingcong Wang

**Affiliations:** 1 Department ICU of the First Affiliated Hospital of Zhejiang Chinese Medical University, Hangzhou, 310006, China; 2 Department emergency of Tianjin medical university general hospital, Tianjin, China

**Keywords:** LC3-II, Beclin-1, autophagy, cerebral ischemia, ischemic post-conditioning

## Abstract

**Objective:**

To investigate the effects of postconditioning ischemia on the expressions of the hippocampus neuron autophagy-related proteins LC3-II and Beclin-1 in rats following cerebral ischemia reperfusion.

**Methods:**

A total of 128 male Sprague–Dawley rats were randomly divided into 4 groups: control, cerebral ischemia-reperfusion (IR), cerebral ischemia post-conditioning group (IP), and PI3K/Akt inhibitor (LY294002). The rat cerebral ischemia model was established by the improved Pulsinelli four vessel occlusion method. The durations across the platform and escape latent period were recorded using the water maze experiment. The changes in cell morphology and the number of surviving hippocampal neurons were detected by hematoxylin-eosin (HE) staining. The cells with Beclin-1 and LC3-II in the hippocampal region were detected by immunohistochemical staining and Western blotting.

**Results:**

When compared with the IR at 48 and 72 h, the number of platform passes increased and the escape latency time was shortened. Consequently, the HE staining detected positive cells with LC3-II and Beclin-1 increased in number at each time point in immunohistochemistry and the expressions of the LC3-II and Beclin-1 proteins were improved in the IP (P < 0.05).

**Conclusions:**

Cerebral ischemic post-conditioning promoted the expressions of autophagy-related proteins LC3-II and Beclin-1 while relieving the injuries caused by cerebral ischemia reperfusion.

Ischemic stroke (cerebral infarction) is a common and frequently occurring cerebrovascular disease, which accounts for 60–80% of all stroke cases. It exhibits the characteristics of high incidence, high morbidity, and high mortality, causing heavy burdens to families and society. Therefore, incidences of ischemic stroke have been widely studied by researchers [1, 2]. Surprisingly, Murry et al. demonstrated that the treatment of multiple short duration ischemia prior to its onset in tissues significantly improved the tolerance of ischemic tissues and also alleviated the ischemic injuries of the tissues [3]. This phenomenon, known as ischemic preconditioning (IPC), has been shown to be an effective measure for mitigating the ischemia/reperfusion (IR) injuries. However, since the preconditioning is performed before ischemic injury, and the tissue ischemia cannot be predicted in advance, the accurate prediction of the most effective time window is rather difficult. Therefore, in 1996, a concept [4] was first proposed by Zhao et al. involving the post-conditioning of ischemia. Ischemic post-conditioning (IPostC) refers to the treatment of the ischemic organ with transient IR before reperfusion. Currently, ischemic post-conditioning is known to reduce a large amount of organ IR injuries, such as damage to the heart, brain, kidneys, and lungs. The protective mechanism involves a variety of endogenous trigger factors and receptors, intracellular signal transduction, and end effectors, which ultimately inhibit the cell apoptosis and necrosis, including trigger-signal pathways [5, 6]. Furthermore, the correlation between autophagy and nervous system diseases has been gradually revealed by several studies [7].

Ischemic post-conditioning is a novel brain protective mechanism which can significantly alleviate the cerebral IR injuries. However, the underlying mechanism has not yet been confirmed, and the relationship with autophagy has been minimally reported. Therefore, the present study investigated the brain protective mechanism of ischemia post-conditioning with respect to autophagy.

## 1 Materials and methods

### 1.1 Experimental materials

A total of 128 healthy male Sprague–Dawley (SD) rats weighing between 300 and 350 g were provided by the Medical Laboratory Animal Center of the North China University of Science and Technology; the SCXK (Beijing) number was 2009-003. The primary rabbit anti-mouse LC3-II and Beclin-1 antibodies and sheep anti-rabbit secondary antibody were obtained from the Beijing Boosen Biological Technology Co. Ltd(Beijing, China). The DAB Kit was obtained from the Zhongshan Golden Bridge Co., while the BCA protein estimation assay kit was obtained from the Beyotime Biotechnology Research Institute and the PI3K/Akt inhibitor (LY294002, from Sigma, Cat# L9908-1MG).

### 1.2 Grouping and handling of laboratory animals

The animals were divided into the control operated group (control) and the cerebral ischemia model group; the latter was further divided into the cerebral ischemia reperfusion group (IR), cerebral ischemia post-conditioning group (IP), and PI3K/Akt inhibitor (LY294002) group. A total of 128 rats were divided into 32 animals in each group. The rats were about 2 months old. Also, 8 rats were allocated to each time point, of which, 4 were used for hematoxylineosin (HE) staining and immunohistochemistry and 4 for Western Blotting.

In the case of control, only the scalp skin was incised without coagulation, and the total catgut embedding was not clamped. In the cerebral ischemia model group, the global cerebral ischemia rat model was constructed using a modified Pulsinelli four vessel block method. The rats were anesthetized using isoflurane. Subsequently, the scalps were cut along the midline occipital, followed by the separation of the paraspinal muscles and fascia, which exposed the first cervical spinous processes. After the first cervical vertebra-shaped holes were visible, the bilateral vertebral arteries were subjected to electrocoagulation. Then, the incisions were disinfection and sutured. The skin was cut along the midline of the necks, and the neck muscles and fascia were separated layer-by-layer, following which, the bilateral common carotid arteries (CCA) were embedded and preserved. After 24 h of postoperative recovery, non-invasive arteries were used for clamping the double CCA for 20 min. The model was successfully evaluated with the following characteristics: coma, righting reflex loss, bilateral mydriasis, and pale eye color. The rats in the IP group were treated with transient IR prior to the complete recovery of reperfusion. The treatments were carried out for 15s each time and repeated three times. Then, the rats were euthanized at the corresponding time points and harvested as specimens. 30 min before the model preparation, cranial drillings were conducted on the LY294002 inhibitor group, and lateral ventricles were injected with 14.92mg LY294002 in 1 mL sterile saline at a final concentration of 100 nmol/μL, 5 μL/each.

**Ethical approval**: The research related to animals use has been complied with all the relevant national regulations and institutional policies for the care and use of animals. The content and methods of this experiment were approved by the Animal Ethics Review Board of the North China University of Science and Technology, and ARRIVE Guidelines were followed for animal studies.

### 1.3 Lateral ventricle injection

The rats were anesthetized using isoflurane after the rats were weighed. The rats were placed in an anesthesia box. The anesthesia box inlet was connected to an anesthesia machine equipped with an isoflurane volatilization tank, and the outlet is connected with the anesthesia monitor and the absorption tank. The isoflurane concentration was maintained at 1.4% and the oxygen flow rate was 2 lpm. The concentration of end-tidal isoflurane in mice was monitored using a gas detector. After continuous isoflurane exposure, the inhalation of isoflurane was stopped and the mice were placed in a thermostat. After a few minutes, the movement of the rats ceased, which was accompanied by slow and deep breathing, limp body characteristics, and disappearance of the light reflexes of the corneal reflection, indicating successful anesthesia.

The whole surgical procedure was carried out under aseptic conditions. The anesthetized rats were fixed on a rat stereotaxic apparatus. The surgical site was routinely shaved and prepared, and then the local surgical area disinfected and the sterile towel covered. The scalp, muscle, and fascia electrocoagulation vertebral artery of the anesthetized rats were sequentially separated. Following a disinfection process and paving with towels, the layers were incised, and the skull cross joints exposed. The locations 0.92 mm in front of the cross and 1.5 mm laterally positioned were selected as the marks. Then, holes were drilled with a clean 5 mL syringe needle that was inserted to 3.5 mm depths with a microinjector, allowing a slow injection of 5 μL LY294002. Subsequently, the needles were maintained in the lateral ventricles for 10 min before pulling out slowly. The wounds were sutured after disinfection. The preliminary experiments were carried out by intracerebroventricular ink injections in order to test the accuracy of localization.

### 1.4 Water maze behavioral testing

A water maze was placed in a well-lit area of the laboratory. A drum with a 120 cm diameter and 70 cm height was used in the experiment. The water depth was 25 cm, and the water temperature was controlled between 22 and 24 °C. A platform 23 cm tall was placed in the middle of the third quadrant, with an 8 cm diameter, 2 cm underwater. During the experiment, the reference was maintained in the appropriate position. Prior to model establishment, all the experimental rats were tested and trained in the navigation experiment for 3 days, with two sessions per day (one in the morning and one in the afternoon). The rats were placed in the four quadrants in order. The time points at which the rats successfully climbed onto the platform within 90 s (escape latency) were tracked systematically and recorded. On day 4, the platform hidden in the water was removed. The rats were placed in the first quadrant, and the number of times the rats crossed the platform was recorded. These tests were not included in the statistical analysis. Following a successful establishment of the model, the above processes were repeated, and the numbers of the 4-time platform-crossing and escape latencies were recorded. Also, an average value was utilized for the statistical analysis.

### 1.5 HE staining

Four rats from each group were randomly selected and anesthetized with the ether. After perfusing with 4% paraformaldehyde (40 g/1000 mL) and fixation, the brain tissues were removed (on ice), along with the intercept brain tissue between the optic chiasm and the brain transverse. After fixation in 4% polyformaldehyde solution for 24 h, the tissues were paraffin-embedded, and the continuous 5 μm-thick coronal sections were sliced. The HE staining was completed following oven baking at 60 °C for 24 h. Subsequently, the sections were dewaxed and dehydrated, stained by hematoxylin for 5–10 min, differentiated by 1% ethanol hydrochloride for 1-2 s, and stained by a 0.5% eosin solution for 1–3 min. Then, the sections were dehydrated with alcohol gradient and treated with xylene to render the slices transparent; the sections were fixed and sealed with a neutral gum. The results were as follows: the cell structures of the living neurons were observed to be normal and arranged neatly and closely; the nuclei were large and round; the staining was homogeneous. Furthermore, four visual fields were observed without overlap at high magnification. The number of normal cells in the hippocampus was measured using a Motic6.0 digital medical image analysis system. Also, the average of the cell number was analyzed statistically.

### 1.6 Immunohistochemical staining

In this study, the sections were dewaxed conventionally. Then, a 3% hydrogen peroxide solution was applied for 10 min to block the endogenous peroxidase activities at room temperature in the dark. The antigen retrieval was carried out at high pressures or in the microwave. Next, the sections were incubated with rabbit anti-rat LC3-II and Beclin-1 antibodies (1:200) in a humidified chamber at 4 °C overnight. After rewarming, the sheep anti-rabbit secondary antibody was added for 1 h at 37 °C. Subsequently, the sections were developed with DAB for 10 min, and restained with hematoxylin for 2-3 min. Following differentiation, full back to blue, and dehydration, the sections were made transparent and sealed. Between each step, a phosphate buffered saline(PBS) buffer wash was applied for 5 min each three times. The primary antibody was replaced with PBS that served as a negative control.

The determination of the results was as follows: LC3-II and Beclin-1 proteins were expressed in the cytoplasm as brown staining with blue nucleus; four slices were selected from each animal; in a high-power magnification (10 × 40), five fields were observed in each slice; a grid eyepiece test system was employed; the positive cells were counted as those with brown granules and a nucleus; and the average value was used for the statistical analysis.

### 1.7 Western blot assay

Following the successful establishment of the model, the rats were sacrificed directly at the corresponding time points. The hippocampus was isolated, placed on ice, and lysed in RIPA buffer. Bradford assay was employed for the protein quantification, followed by standard sodium dodecyl sulfate-PAGE（SDS-PAGE）. The proteins were transferred to Polyvinylidene Fluoride (PVDF) membrane and blocked with skim milk in Tris-buffered Saline with Tween (TBST). Subsequently, the membranes were probed with primary antibodies (LC3-II and Beclin-1 at 1:800; β-actin 1:1000) overnight at 4 °C. Then, the membrane was washed with TBST and incubated with the secondary antibodies (1:2000) for 1 h at room temperature, followed by detection of the immunoreactive bands with ECL (Bio-Rad gel imaging analysis system). The protein levels were expressed by the absorbance ratio of the target band and β-actin as the reference.

### 1.8 Statistical analysis

In this study, a database was directly established using IBM SPSS 17.0 software (IBM SPSS, Armonk, NY, USA) for the statistical analysis. The continuous variables with normal distributions were expressed as either mean *x* ± *s* or median (quartile). A single factor analysis of the variance was used comparatively, as well as a repeated measure analysis of the variance. Levene was utilized to test the homogeneity of the variance. Following the analysis of a one-way ANOVA homogeneity of variance, the least significance difference (LSD) and post-hoc tests were employed. When the variance was found to be uneven, a Games–Howell test was utilized. The significant differences were indicated as *P* < 0.05.

## 2 Results

### 2.1 Water maze test results for the rats in each group

**Figure 1:**
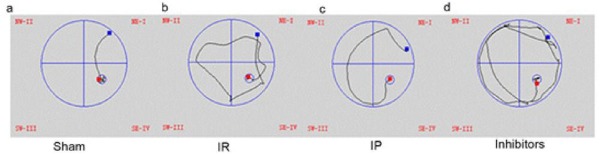
Swimming trajectories of rats in each group at 48 h after the water maze test.

In this study, the frequency of the platform crossings in the cerebral ischemia group was found to be reduced in the water maze, and the escape latency times were prolonged when compared with the control operational group (*P* < 0.05). Also, in comparison with the cerebral ischemia group, the platform crossing duration in the water maze was increased in the ischemia post-conditioning group, and the escape latency times were shortened (*P* < 0.05). Furthermore, the frequency of the platform crossings in the inhibitor LY294002 group was determined as reduced and the escape latency times were prolonged as compared to the ischemic post-conditioning group (*P* < 0.05). The results are shown in Table 1 and Figure 1.

**Table 1:** Comparison of the times across platform and escape latent period of rats in each group by the water maze test ( *x* ± *s* ) (*n* = 8)

Group	Times across platform		Escape latent period	
	
	48 h	72 h	48 h	72 h
Sham group	12.43 ± 0.19	12.18 ± 0.21	20.43 ± 0.29	20.18 ± 0.21
IR group	2.36 ± 0.65^*^	1.75 ± 0.55^*^	44.55 ± 2.65^*^	50.79 ± 1.78^*^
IP group	4.61 ± 0.71^*#^	3.36 ± 0.57^*#^	37.41 ± 1.81^*#^	45.02 ± 2.12^*#^
LY294002 group	2.12 ± 0.45^Δ^	1.62 ± 0.39^Δ^	46.12 ± 1.77^Δ^	52.64 ± 2.01^Δ^

*Note*: *Compared with the sham group, *P* ＜ 0.05; #compared with the IR group, *P* ＜ 0.05; ^Δ^compared with the IP group, *P* ＜ 0.05.

### 2.2 Results of HE staining of the rats in each group

The structures of the neurons in the hippocampus of the control group rats at different time points were determined to be normal and arranged in an orderly manner. The nuclei were large and round, and the nucleoli were prominent. Also, the staining was uniform, and the structures complete. However, the structures of the nerve cells in the hippocampus of the cerebral ischemia rats were found to be damaged. Moreover, after 6 h, the neural cells in the hippocampus showed partial hyperchromatic staining. Furthermore, then nucleus deformation boundary was not distinct. After 24 h, the visible nerve cells of the nuclei showed significant deformations, accompanied by pyknosis, pronounced staining, interstitial edema, loose structures, and partial cytoplasmic autolysis.

**Figure 2:**
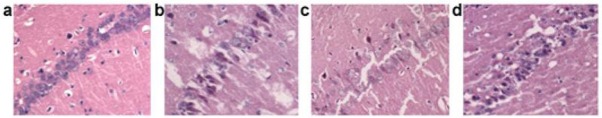
Hematoxylin and eosin (HE) staining result of rat hippocampal neurons at 24 h in each group (HE 10 × 40). a: Control operation group; b: Cerebral ischemia group; c: Ischemic post-conditioning group; d: Inhibitor group.

At the 48- and 72-h time points, the nerve cell damage was found to increase continually and was accompanied by a large number of vacuoles, disappearance of the nucleolus, nuclear pyknosis, and fragmentation. In comparison to the ischemic group, the damages to the neuronal structures in the hippocampus showed improvement in the ischemic post-conditioning group, which was accompanied by decreased interstitial edema, and reduced number of vacuolar structures. Furthermore, the neuron damages were aggravated in the inhibitor LY294002 group. In comparison to the control operational group, the number of surviving neurons decreased significantly in the cerebral ischemia group at each time point. Moreover, the expression decreased at 6, 24, 48, and 72 h; the maximal surviving neuronal cells reached the maximum at 6 h. The number of surviving neurons at different time points increased significantly in the ischemia post-conditioning group as compared to the cerebral ischemia group. Also, the number of surviving neurons was observed to be reduced at different time points in the inhibitor LY294002 group as compared to the ischemia post-conditioning group (Table 2 and Figure 2).

**Table 2:** Number of surviving neurons in the rat hippocampus in each group (*x* ± *s* ) (*n* = 4)

Group	6 h	24 h	48 h	72 h
Sham group	132.00 ± 1.90	132.50 ± 1.37	132.83 ± 2.23	132.83 ± 2.13
IR group	75.17 ± 1.94^*^	87.50 ± 2.59^*^	96.00 ± 2.19^*^	103.17 ± 1.94^*^
IP group	87.00 ± 2.83^*#^	103.17 ± 1.47^*#^	109.83 ± 2.31^*#^	114.33 ± 2.58^*#^
LY294002 group	61.83 ± 1.47^Δ^	76.50 ± 2.35^Δ^	84.50 ± 1.38^Δ^	92.33 ± 2.42^Δ^

*Note*: *Compared with the sham group, *P* ＜ 0.05; #compared with the IR group, *P* ＜ 0.05; ^Δ^compared with the IP group, *P* ＜ 0.05.

As observed in the image, the cell structures of the control operated group were normal. The neuron structures exhibited different degrees of damage in the cerebral ischemia, ischemia post-conditioning, and inhibitor groups. In comparison to the Ischemic group, the structures were improved in the ischemic postconditioning group, and the number of surviving neurons increased. Furthermore, in comparison to the ischemia post-conditioning group, the structural damages appeared to be aggravated in the inhibitor group, and the number of surviving neurons were reduced.

### 2.3 LC3-II immunohistochemical staining results of the rats in each group

**Figure 3:**
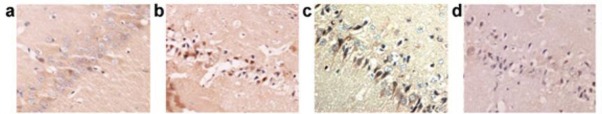
Immunohistochemical staining results of LC3-II in rats’ hippocampus at 24 h in each group (IHC 10 × 40). a: Control operational group; b: Cerebral ischemia group; c: Ischemic post-conditioning group; d: Inhibitor group.

The LC3-II was expressed as puncta (that were aggregated on the membrane of the autophagic body). In the control group, of LC3-II was weakly expressed in the cytoplasm. However, the other two groups showed different degrees of positive expressions. The expression of LC3-II in the neuron cells increased at each time point in the cerebral ischemia group as compared to the control operational group, and the changes in the time expression changes were as follows: the positive expression of LC3-II at 6h time point began to increase and peaked at 24 h. Eventually, the expression decreased at 48- and 72-h time points. Moreover, the LC3-II expression in the neuron cells increased at each time point in the ischemia postconditioning group as compared to the cerebral ischemia group. In addition, the dynamic expression changes at each time point were similar to that in the cerebral ischemia group. The number of LC3-II positive cells in the hippocampus decreased at each time point in the inhibitor LY294002 group as compared to the ischemia post-conditioning group (Table 3 and Figure 3).

**Table 3:** Number of LC3-II-positive cells in the rat hippocampus in each group (*x* ± *s* ) (n = 4)

Group	6 h	24 h	48 h	72 h
Sham group	4.02 ± 0.33	4.67 ± 0.23	4.27 ± 0.12	4.33 ± 0.21
IR group	17.87 ± 0.43^*^	24.54 ± 0.49^*^	15.34 ± 0.72^*^	13.76 ± 0.71^*^
IP group	24.30 ± 1.12^*#^	30.30 ± 1.36^*#^	22.09 ± 1.25^*#^	17.37 ± 1.65^*#^
LY294002 group	15.23 ± 0.79^Δ^	21.34 ± 1.12^Δ^	12.56 ± 0.55^Δ^	11.21 ± 0.64^Δ^

*Note*: *Compared with sham group, *P* ＜ 0.05; #compared with the IR group, *P* ＜ 0.05; ^Δ^compared with the IP group, *P* ＜ 0.05.

Furthermore, the expression of LC3-II in the cerebral ischemia and ischemic post-conditioning groups was higher than that in the control operational group. Also, the expression in the ischemic post-conditioning group reached the maximum.

### 2.4 Immunohistochemical staining results of Beclin-1 in each group

**Figure 4:**
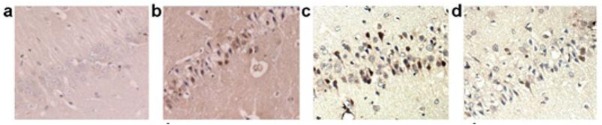
Immunohistochemical staining results of Beclin-1 in rats’ hippocampus at 24 h of each group (IHC 10 × 40). a: Control operational group; b: Cerebral ischemia group; c: Ischemic post-conditioning group; d: Inhibitor group.

In the control group, Beclin-1 was found to be weakly expressed in the cytoplasm, whereas the other two groups showed different degrees of positive expression of the protein. In the cerebral ischemia group, the Beclin-1-positive cells in the hippocampus increased in number at different time points as compared to the control operation group. The expression increased after 6 h and peaked at 24 h. Moreover, at the 48- and 72-hour time points, the expression decreased gradually. In comparison to the cerebral ischemia group, the number of Beclin-1-positive cells in the hippocampus increased at each time point in the ischemia post-conditioning group. Also, the dynamic expression changes at each time point were similar to those in the cerebral ischemia group. In the inhibitors LY294002 group, the number of Beclin-1-positive cells in the hippocampus was reduced at different time points in comparison to the ischemia post-conditioning group (Table 4 and Figure 4).

**Table 4:** Number of Beclin-1-positive cells in the rat hippocampus of each group (*x* ± *s* ) (*n* = 4)

Group	6 h	24 h	48 h	72 h
Sham group	2.12 ± 0.27	2.52 ± 0.63	2.33 ± 0.62	2.50 ± 0.37
IR group	12.79 ± 1.25^*^	16.50 ± 1.18^*^	10.20 ± 1.40^*^	7.39 ± 1.71^*^
IP group	16.24 ± 1.17^*#^	22.26 ± 1.47^*#^	14.55 ± 0.99^*#^	12.79 ± 1.38^*#^
LY294002 group	10.22 ± 0.89^Δ^	14.11 ± 1.23^Δ^	7.34 ± 0.78^Δ^	5.45 ± 1.01^Δ^

*Note*: *Compared with the sham group, *P* ＜ 0.05; #compared with the IR group, *P* ＜ 0.05; ^Δ^compared with the IP group, *P* ＜ 0.05.

Furthermore, the expression of Beclin-1 in the cerebral ischemia and ischemic post-conditioning groups was higher than that in the control operational group, while maximal expression was observed in the ischemic postconditioning group.

### 2.5 LC3-II protein expression in the hippocampus of the rats in each group

**Figure 5:**
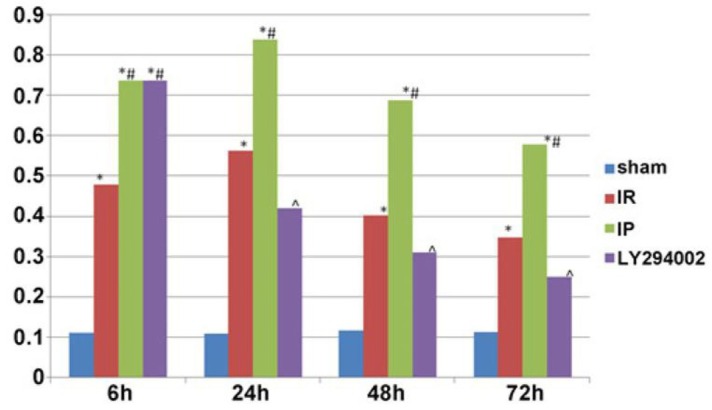
Western Blotting results of LC3-II protein in the hippocampus of each group: *P<0.05 compared to the Control; ^#^*P<*0.05 compared to the IR; ^*P<*0.05 compared to the IP.

**Figure 6:**
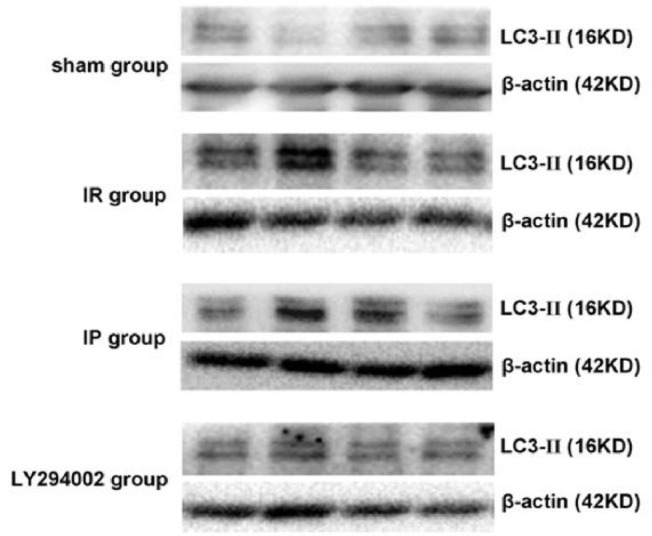
Expressions of the LC3-II protein in the hippocampus in each group assessed by Western Blotting analysis.

The relative expression of the LC3-II protein was presented as the ratio of LC3-II /β-actin optical density value. A small amount of the LC3-II protein expression in the hippocampus was observed in the control operational group at different time points. In the cerebral ischemia group, the expression of the LC3-II protein in the hippocampus was determined to be increased as compared to the control operational group at the different time points. At the 6-h time point, the expression increased and maximized at the 24-h time point. Then, at 48- and 72-h time points, the expression decreased gradually (*P* < 0.05). Moreover, the expression of the LC3-II protein increased in the hippocampus at each time point in the ischemia postconditioning group as compared to the cerebral ischemia group. The dynamic expression changes at each time point were found to be identical to that of the cerebral ischemia group (*P* < 0.05). The LC3-II protein expression in the hippocampus decreased at the different time points in the inhibitor LY294002 group as compared to the ischemia post-conditioning group (*P* < 0.05) (Figs. 5 and 6).

### 2.6 Beclin-1 protein expression in hippocampus of the rats in each group

**Figure 7:**
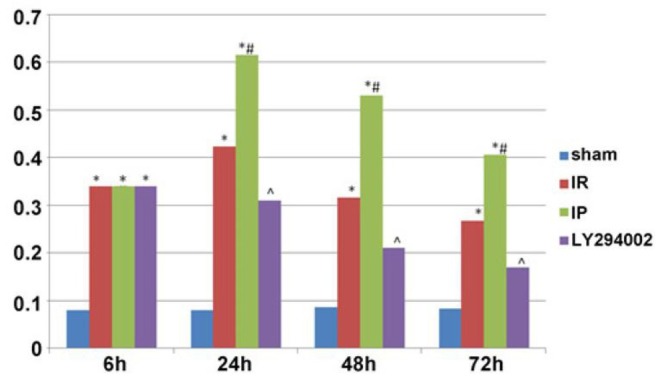
Western blotting results of the Beclin-1 protein in the hippocampus of each group: *P<0.05 compared to the Control; #*P<*0.05 as compared to the IR; ^*P<*0.05 compared to the IP.

**Figure 8:**
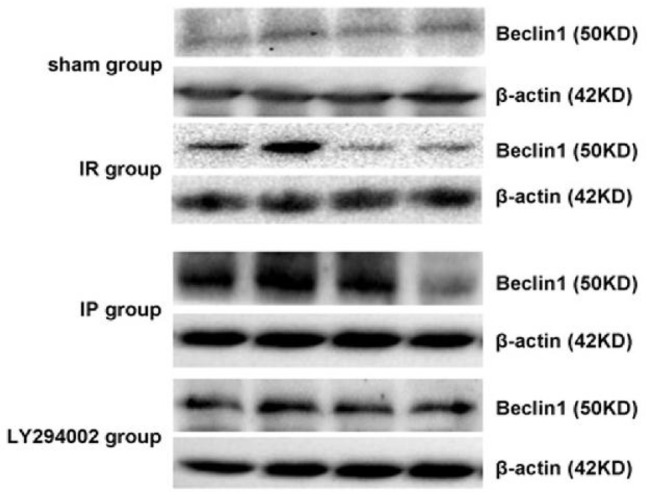
Expression of the Beclin-1 protein in the hippocampus in each group tested by Western Blotting analysis.

The relative expression of the Beclin-1 protein was presented as the ratio of the optical density value of Beclin-1/β-actin. A small amount of Beclin-1 protein in the neuron cells was expressed at each time point in the control operational group. Also, the protein was found to be increased in the hippocampus at different time points in the cerebral ischemia group as compared to the Control group; the expression increased after 6 h and peaked at 24 h. Furthermore, at the 48- and 72-h time points, the expression decreased gradually (*P* < 0.05). Moreover, the protein expression increased in the hippocampus at each time point in the ischemia post-conditioning group as compared to the cerebral ischemia group, and the dynamic expression change at each time point was determined to be identical to that in the cerebral ischemia group (*P* < 0.05). In comparison to the ischemia post-conditioning group, the Beclin-1 protein expression was found to be decreased at different time points in the hippocampus of the inhibitor LY294002 group (*P* < 0.05) (Figs. 7 and 8).

## 3 Discussion

In the current study, the cerebral ischemia reperfusion rat model was established using a modified Pulsinelli four vessel block (4-VO) method, and the duration of ischemia was 20 min. In the ischemic post-conditioning group, ischemia 15s/reperfusion 15s was repeated three times before the complete recovery of reperfusion. The changes in the learning and memory abilities of each group were examined by the water maze tests, and the pathological changes in the neurons in the hippocampus of the rats were observed by HE staining. The comparison of the cerebral ischemia and ischemic post-conditioning groups revealed that the number of platform crossings increased at the 48- and 72-h time points after ischemia post-conditioning. Also, the escape latency was found to be significantly shortened. At 6, 24, 48, and 72 h after ischemia post-conditioning, the HE staining of the neuronal cell damages was improved significantly, and the survival rate of the neurons increased significantly. These findings indicated that the cerebral ischemia post-conditioning could potentially improve the nerve dysfunctions and reduce the neuronal cell damages and deaths of rats caused by cerebral ischemia reperfusion, resulting in remarkable neuroprotective effects. These results were found to be consistent with those obtained from previous studies.

Ischemic post-conditioning refers to the treatment of the ischemic organ with transient ischemia/reperfusion before reperfusion. The initial research on ischemic post-conditioning was focused on myocardial ischemia reperfusion injuries. In 2003, Zhao et al. first confirmed that myocardial ischemic post-conditioning alleviated the myocardial ischemia reperfusion injuries due to its endogenous protective effects [8]. Currently, ischemia postconditioning has been confirmed clinically in the cardiac field. Laskey et al. [9] selected 30 patients with myocardial infarction percutaneous coronary angioplasty as a study group and conducted four brief ischemia/reperfusion balloon deflation post-conditioning procedures before reperfusion, while the control group received direct reperfusion. The results showed that the myocardial enzyme peak decreased in the study group as compared to the control group. Also, the amplitude of the ST segment increased, and the abnormal motion area was reduced. In recent years, extensive studies have been conducted regarding ischemic brain post-conditioning. The results of these studies have proved that ischemic post-conditioning also exerts endogenous protective effects. Wang et al. [10] confirmed that if after 10-min cerebral ischemia, multiple transient reperfusions/ischemic treatments were administered immediately, the neuron cell damages and necrosis in the hippocampus and parietal brain skin layer after reperfusion for 7 days were reduced significantly. In addition, the present study determined that the spatial memory and learning abilities of the rats were enhanced in the water maze tests after reperfusion for 3weeks.

Intriguingly, few studies reported the autophagy after cerebral ischemic postconditioning [11, 12]. Hu et al. [11] demonstrated that the levels of Beclin-1 and LC3 proteins in the cortex were upregulated following treatment with repeated limb remote ischemic post‑conditioning using an endovascular puncture rat model of subarachnoid haemorrhage (SAH). Wang et al. [12] found that cerebral ischemic postconditioning inhibited autophagy and high mobility group box 1 (HMGB1) secretion; autophagy inhibition induced a decrease in HMGB1 secretion, and attenuation of HMGB1 secretion inhibited autophagy, as evaluated by immunofluorescence and Western blotting; however, the oxygen glucose deprivation cellular model was utilized in the study.

The microtubule associated protein LC3 (MAP-LC3) is known as the analogue of the autophagy-related gene *Atg8* in yeast cells. The LC3-II/LC3-I complex is a critical index for assessing the levels of autophagy [13]. Beclin-1 is a homologue of yeast ATG6, a member of the Bcl-2 family and a component of the PI3K complex; it was one of the primary topics of research on autophagy-related protein in previous studies. The results showed that Beclin-1 bound to a variety of proteins, such as Vps34 (ClassIIIPI3K catalytic subunit), mTOR, Bcl-2, and BCLXL. In addition, it participated in autophagy regulation, and was designated as the autophagy regulatory gene [14]. Furthermore, since autophagy is regulated by PI3K/Akt/mTOR signaling pathway [15], we selected PI3K/Akt inhibitor (LY294002) as a control to explore autophagy after PI3K/Akt inhibition. Moreover, LY294002 was confirmed to inhibit LC3-II and Beclin-1. Autophagy occurs in various tissue cells and can be activated under different conditions, such as cell nutrient deficiency, hypoxia, and ischemia. It degrades the damaged organelles and protein in order to provide amino acids, fatty acids, and other substances for cell survival. Intriguingly, autophagy can be activated rapidly and is involved in cell death during cerebral ischemia reperfusion. The environment around the cells, as well as, the progression stage of the disease, determines the role of autophagy. Wen et al. [7] determined that autophagy was activated after ischemia, resulting in a large number of autophagosomes in the visible brain cells. Subsequent studies demonstrated that the administration of inhibitors against 3-Methyladenine (3-MA) and cathepsin significantly reduced the cerebral infarction area, as well as, improved the structure of the nerve cells. These findings illustrated that the autophagy induced by ischemia may aggravate brain injuries. In this study, the expression of LC3-II and Beclin-1 in the cerebral ischemia group was found to be significantly increased after 6 h, peaked at 24 h, and decreased at the 48- and 72-h time points. However, the expression of these proteins was still higher than that in the control operational group, cementing the occurrence of cerebral ischemia-activated autophagy, in agreement with the results of previous studies.

Nevertheless, the mechanism underlying the effects of ischemic post-conditioning on autophagy is yet to be elucidated. One possibility is that autophagy might be involved in the protection of the brain from ischemia postconditioning. In this study, the autophagy expression after ischemia post-conditioning was tested. In the ischemic post-conditioning group, the LC3-II and Beclin-1 expressions at the different time points were significantly higher than those of the cerebral ischemia group. Therefore, when combined with the results of the HE staining, a high expression of autophagy is speculated as one of the endogenous protective mechanisms underlying cerebral ischemia post-conditioning.

The results showed that the cells could initiate the programmed cell death processes under different environmental conditions. In addition to the apoptosis of cathepsin and lysosomal hydrolases, autophagic programmed deaths, independent of cathepsin and lysosomal hydrolases, were observed [16]. Moreover, autophagy and apoptosis occurred simultaneously and also interacted with each other. The apoptotic pathway could potentially promote autophagy activation after it was inhibited. Furthermore, moderate autophagy could remove apoptotic residues and alleviate tissue damages. On the other hand, excessive activation of autophagy in the apoptosis pathway might not be sufficient to alleviate the damages. Therefore, the apoptosis in the body cannot be cleaned promptly, which would putatively aggravate the inflammatory reactions and tissue damages. The excessive activation of autophagy causes cell death autophagic procedures, which could potentially trigger the apoptosis pathway [17]. Espert et al. [18] found that during the examination of spinal cord ischemia reperfusion injuries, ischemia post-conditioning displayed an enhanced protection role of the activity of autophagy at the reperfusion stage. The mechanism might include ischemic post-conditioning-mediated promotion of the phosphorylation of Bcl-2 and Beclin-1/Bcl-2 complex separation, as well as, enhanced autophagy expression. Moreover, Bcl-2 is an anti-apoptotic factor, indicating that the ischemia post-conditioning promoted cell survival by regulating the autophagy and apoptosis.

Nevertheless, the limitation of this study was the detection of only Beclin-1 and LC3. Thus, further investigation of the expression of autophagy-related mTOR and P62 proteins in the brain tissue is essential.

In conclusion, cerebral ischemic post-conditioning promoted the expression of autophagy-related proteins, LC3-II and Beclin-1, while relieving the injuries caused by cerebral IR.
